# Using Real-Time Coronial Data to Detect Spatiotemporal Suicide Clusters: A Feasibility Study

**DOI:** 10.1027/0227-5910/a000968

**Published:** 2024-08-13

**Authors:** Leo Roberts, Angela Clapperton, Jeremy Dwyer, Matthew J. Spittal

**Affiliations:** ^1^ Melbourne School of Population and Global Health, The University of Melbourne, Parkville, VIC, Australia; ^2^ Coroners Prevention Unit, Coroners Court of Victoria, Melbourne, VIC, Australia

**Keywords:** **s**uicide clusters, real-time registers, surveillance, scan statistic, SaTScan

## Abstract

**Abstract:** *Background:* Real-time suicide registers are being established in many countries and enable regular monitoring of suspected suicides over time. The use of these data to monitor for suicide clusters is in its infancy. *Aims:* We sought to test the feasibility of using real-time suicide register data to detect spatiotemporal suicide clusters. *Method:* Using the Victorian Suicide Register and SaTScan’s spatiotemporal scan statistic, we simulated a monthly search for clusters from January 2015 to June 2022 using rolling 2-year windows of data in each search. Monthly scans were performed at three different levels of geographic granularity and for all-ages and under-25 populations. *Results:* Our results indicated the rapid identification of possible suicide clusters and demonstrated a practical approach to combining real-time suicide data and scanning algorithms. We developed new model outputs that showed cluster timelines. *Limitations:* The main limitations are that the computational burden of fitting multiple models meant we were unable to scan for ellipses and other irregular shapes and we were unable to consider space–time permutation models. *Conclusions:* Using data from a real-time suicide register, we were able to scan for space–time suicide clusters simulating the situation where the data are updated monthly with new updates.

Suicide is a major societal problem. According to the World Health Organization, there are nearly 700,000 suicides each year. Among those aged 15–29 years, suicide is the fourth leading cause of death (World Health Organization, 2021). Suicides have a particularly devastating impact when they occur in clusters – a group of suicides that occur closer together in time and space than expected given the underlying suicide rate (Hawton et al., 2020). Clusters are of significant concern (a) because they typically occur in groups in which news of a suicide can spread rapidly and widely (e.g., young people) and (b) because of the potential for each suicide in a cluster to lead to further suicides in the community (Hawton et al., 2020; Hill et al., 2020).

Hawton et al. (2020) identified four mechanisms that may explain why clusters occur. One mechanism is social transmission, whereby exposure to suicide or self-harm (either directly through person-to-person exposure or indirectly through the media) leads to suicidal behavior in another individual. A second mechanism, descriptive norms, occurs when an individual believes that suicidal behaviors are more common in the community than they truly are. A third mechanism, assortative relating, refers to a social network of people with pre-existing susceptibilities and where those susceptibilities increase the risk of suicidal behavior within the network. A final mechanism, social integration and regulation, refers to the rapid spreading of information and behaviors in closed communities with high social isolation and high social cohesion. Importantly, Hawton et al. noted that these mechanisms are not mutually exclusive and two or more may operate at once.

In Australia, one of the challenges in detecting suicide clusters has been a reliance on data based on an investigation by a coroner. These investigations are often lengthy – typically 12–18 months – rendering these data too old to be used for detecting suicide clusters as they are occurring. This limits the opportunities for intervention. However, this challenge could be overcome by using data from a real-time suicide surveillance system in which information about suicides is rapidly processed and used to inform decision-making if there is evidence of an emerging cluster. Real-time systems have been established in many countries and use data from police reports and death certificates to identify suspected suicides (Pirkis et al., 2022). These systems produce counts of suicides that correspond closely with those from vital statistics systems (Baran et al., 2021; Leske et al., 2023).

In Victoria, Australia, the Coroners Court of Victoria has maintained the Victorian Suicide Register (VSR) since 2012 (Sutherland et al., 2018). Information about a suspected suicide is added to the register in stages as an investigation proceeds. Core data are added within 24–48 h of police reporting a suspected suicide to the court. This includes basic demographic information, information on the suicide method, and geocoordinates for the location of the fatal incident and the deceased’s residential address. Since information about a suspected suicide is entered into the register soon after death, the VSR effectively captures suspected suicides in real time. As such, the VSR resolves the historical problem of timeliness that has hampered suicide cluster surveillance in Victoria and other jurisdictions. Access to geocoordinates in the VSR also resolves another issue that has impeded suicide cluster surveillance – the modifiable area unit problem (Wong, 2009). This problem refers to the risk that results are influenced by the choice of geographic unit used in the analysis (i.e., the geographic unit that suicides are aggregated up to prior to scanning for clusters).

In this study, we sought to test the feasibility of using VSR data to detect suicide clusters in real time using a methodology similar to how we envisage cluster detection being done in practice. This involved simulating an implementation of real-time suicide cluster detection using historical VSR data. The results of the analysis are not simulated and reflect suspected historical suicide clusters in Victoria.

## Methods

### Suicide Data

We used VSR data containing information on all suspected suicides between January 1, 2015, and June 30, 2022. The data analyzed in this study comprised the date of the suspected suicide, the deceased’s age at death, and the location of the deceased’s usual residence as latitude and longitude coordinates. Other data provided, but not included in the final analytical models, consisted of the fatal incident location (also as geocoordinates), deceased sex, and suicide method. Residential location was used exclusively for cluster detection analysis, as opposed to incident location. This choice reflected the goal to use real-time data to identify communities in crisis, rather than to identify locations where multiple suicides occurred. Accordingly, we excluded any suspected suicides without Victorian residential address coordinates; these mostly comprised people without permanent places of residence, and interstate and overseas visitors to Victoria.

The VSR is highly accurate at detecting suicides. A 5-year analysis of VSR data found high levels of real-time classification accuracy: Ninety-three percent of deaths initially classified as suspected suicides during real-time surveillance were confirmed to be suicides upon review after the coroner’s investigation was complete. Furthermore, of all cases eventually classified as suicides following completion of the coroner’s investigation, only 5.1% of these were not initially classified as suicide (Dwyer et al., 2021).

### Population and Digital Boundary Data

To account for population density, we gathered population counts at three different spatial levels as defined by the Australian Bureau of Statistics (ABS), where the lowest level aggregated up the next level and so on. We accessed data on the number of usual residents in each mesh block (MB; 30–60 dwellings), statistical area level 1 (SA1, ∼400 people), and statistical area level 2 (SA2, ∼10,000 people) in Victoria for all ages and for people aged <25 years. All population counts were based on the 2016 Australian Census. We also accessed digital boundary files (shapefiles) for each MB, SA1, and SA2 from 2016 Australian Census collections provided by the ABS.

Using the shapefiles, we performed two procedures necessary for cluster detection modeling. First, we determined which MB, SA1, and SA2 enclosed each pair of coordinates for the deceased via a spatial join to the relevant shapefile. Second, we identified a centroid location for each MB, SA1, and SA2. For SA1s and SA2s, we calculated population weighted centroids using 2016 ABS population estimates from the constituent MBs as weights. Separate calculations were performed for people under 25 years and people of all ages, given different population distributions. For MBs, we calculated the geometric centroid because more finely grained population estimates were not available. All spatial procedures were performed with the R package, *sf* (Pebesma, 2018).

### Analyses

A series of suicide cluster detection analyses were performed with the opensource software, SaTScan (v10.0.2). SaTScan’s spatiotemporal scan statistic evaluates potential clusters by exhaustively moving a window of variable spatial and temporal size across a geographic space, while recording, for each variation of the window, the observed and expected number of events inside and outside the window (Kulldorff et al., 1998). To detect suicide clusters, we applied the discrete Poisson model (in retrospective mode), under which the number of cases in each location was assumed to be Poisson-distributed, according to the population at risk (Kulldorff, 1997, 2022). Given the null hypothesis of equal disease risk, the expected number of cases in each area is proportional to the person-years in that area (Kulldorff, 1997, 2022). To identify a potential cluster, SaTScan calculates a log likelihood ratio for each window (the test statistic) that measures the risk of suicide inside the window relative to the risk outside, which is then maximized across all windows to establish the most likely cluster (Kulldorff, 2022). Monte Carlo simulation tests the significance of the most likely cluster and other high-likelihood clusters by (a) finding the maximum test statistic in 999 random datasets drawn from the space–time landscape provided to SaTScan and (b) ranking the candidate cluster’s test statistic in the set of 1,000, with the *p* value corresponding to the rank (e.g., *p* = 50/1,000 = .05; Kulldorff, 1997).

SaTScan models are subject to several influential parameters. Following extensive pilot testing on a complete historical dataset, we set up SaTScan to search for clusters using circular windows with a size limit of 100 km in radius or dimensions that captured 1% of the population (whichever applied) and a maximum temporal duration of 12 months. Potential clusters were constrained to monthly units as no significant day level clusters were found in pilot testing (i.e., no clusters of less than 1 month). We classified candidate clusters as possible clusters if their *p* value was ≤ .01. This liberal alpha value recognized that occasional false positives would have little downside in a suicide surveillance system (e.g., the ramification might be additional file review in the first instance). Historical clusters identified in pilot testing were consistent with locations suspected by local experts.

To simulate real-time cluster detection, we ran spatiotemporal models over progressively more recent 2-year epochs of suicide data. The first model assessed suicide counts between January 2015 and December 2016 inclusive (i.e., 24 months of data), with the next model assessing from February 2015 to January 2017. This process was repeated until the final model examined suicide counts between July 2020 and June 2022. The simulation was performed at the MB, SA1, and SA2 levels for people of all ages and for people under 25 years. In total, this resulted in the execution and analysis of 402 models (201 for each age group). SaTScan modeling was executed in R (v3.4.4) via the *rsatscan* package (Kleinman, 2015). R scripts were written to prepare the case, coordinate and population files required to execute SaTScan, set SaTScan parameters, run the analysis, and compile and report the results.

At the conclusion of modeling, we collated information about all significant clusters, noting the epoch that *first* revealed each cluster. We considered this date an estimate of when clusters would have been identified in a system where the data were updated with new data, every month.

### Ethical Approval

Ethics approval was obtained from The University of Melbourne’s Human Research Ethics Committee (reference: 2021-21928-20627-3). The Victorian State Coroner signed an order for the release of coronial data for the purpose of this project and an information sharing agreement between the Court Services Victoria (acting on behalf of the Coroner’s Court of Victoria) and The University of Melbourne was executed in October 2021.

## Results

There were a total of 5,106 suspected suicides between January 2015 and June 2022. For 128 records, the deceased did not have a valid Victorian residential address and were excluded from subsequent analyses. This resulted in data on 4,978 deaths (1,294 females, 3,684 males, *M*_age_ = 46 years).

### All-Ages Simulation

Table 1 summarizes the details of the earliest detection of each significant cluster from the all-ages simulation. In total, 16 distinct significant clusters were identified, noting that sometimes technically distinct clusters could be practically mapped to a single cluster (e.g., Cluster 3). The duplication occurred either because separate MB, SA1, and SA2 models homed in on the same set of deaths or because a nearby space–time window also enclosed a significant cluster. In either case, a different cluster boundary was identified even if it was capturing essentially the same group of events.

**Table 1:** Significant clusters identified in the all-ages simulation

Cluster	Geography level	Duration of cluster (months)	Number of areas in cluster	Observed	Expected	*p* value	Population of cluster area
1a	SA2	7	2	11	1.746	.094	26,200
2a	SA2	11	3	17	4.156	.097	40,000
3a	MB	10	34	8	0.257	.006	2,700
3b	MB	9	101	9	0.497	.043	6,000
3c	MB	7	101	8	0.395	.1	6,000
3d	SA1	9	16	9	0.475	.015	6,000
3e	SA1	7	16	8	0.367	.023	6,000
4a	SA2	8	3	15	3.016	.04	40,000
4b	SA2	10	3	17	3.825	.041	40,000
4c	SA2	9	4	17	3.845	.033	44,000
5a	MB	8	220	10	0.749	.086	10,000
5b	SA1	8	27	10	0.859	.089	11,500
6a	MB	1	7	nr	0.001	>.01	>150
7a	SA2	5	6	14	2.825	.08	58,600
8a	MB	8	2	nr	0.003	>.05	>50
8b	SA1	8	1	nr	0.007	>.10	>100
*Note*. Functionally equivalent clusters have been grouped (e.g., 3a–3e), even if they have marginally distinct cluster dimensions. nr = not reported due to small cell sizes. Where this occurs, the *p* value and the population size have been rounded up to preserve the anonymity of the cluster’s location.							

**Figure 1:**
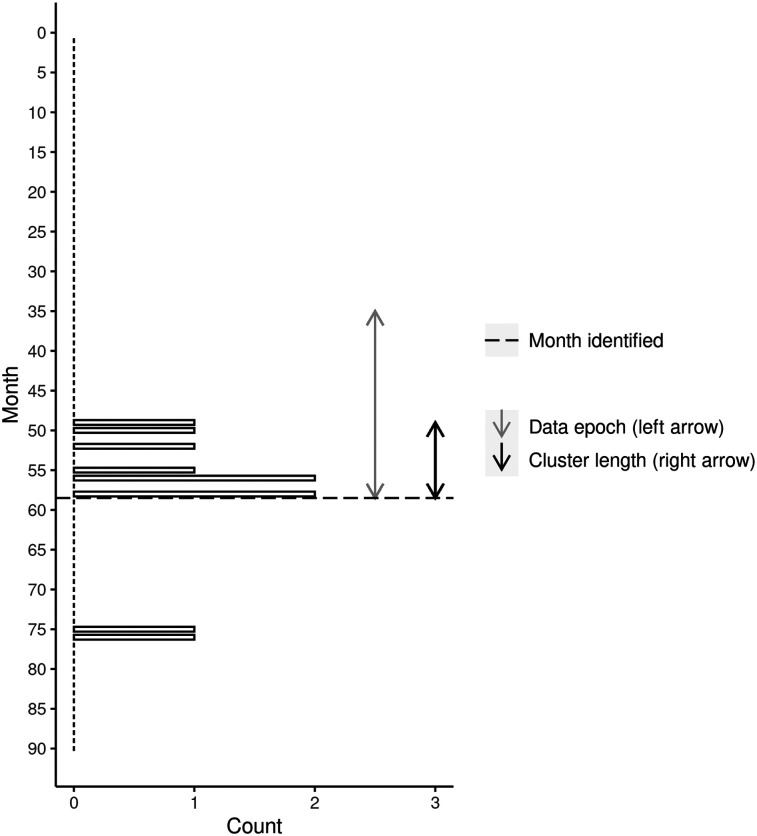
Timeline of cluster detection and suicide events for all-ages cluster 3a (8 suicides).

**Figure 2:**
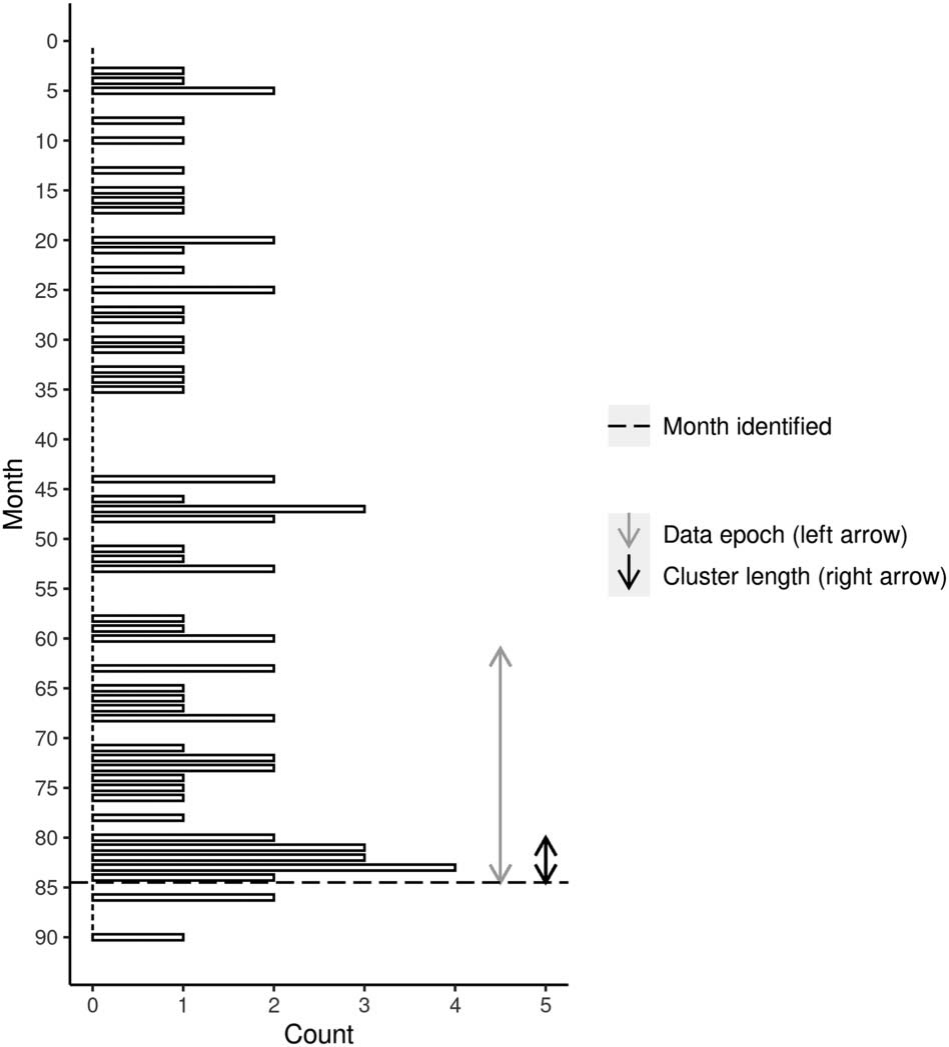
Timeline of cluster detection and suicide events for all-ages cluster 7a (14 suicides).

Figures 1 and 2 display the detection timeline for two example clusters (Cluster 3a and Cluster 7a). These figures indicate (a) the suicide history within the geographical area of the cluster since 2015, (b) the 2-year epoch modeled (left arrow), (c) the cluster duration (right arrow), and (d) when the cluster would have (theoretically) been identified given the monthly provision of suicide data.

Figure 1 demonstrates that Cluster 3a would have been theoretically detected at the end of month 59, immediately following provision of that month’s data. Figure 1 also indicates that before this cluster, suicide was uncommon in the area (no events recorded since January 2015). While the cluster appeared to be detected when effectively over, two further suicides occurred later in the timeline. Timely identification of this cluster might have created a chance for intervention in the area, although whether these two suicides could have been averted is an open question. Figure 2 demonstrates that Cluster 7a would have been theoretically detected at the end of month 85, once again immediately following provision of that month’s data. While this area had a history of suicide before the cluster, a relatively large group of deaths is apparent throughout. Moreover, two further suicides occurred shortly after the estimated cluster detection time, again highlighting the possibility of intervention. Figures 1 and 2 imply that given 2-year epochs of Victorian Suicide data, and a monthly data feed, significant clusters could be rapidly detected.

### Under-25 Simulation

**Figure 3:**
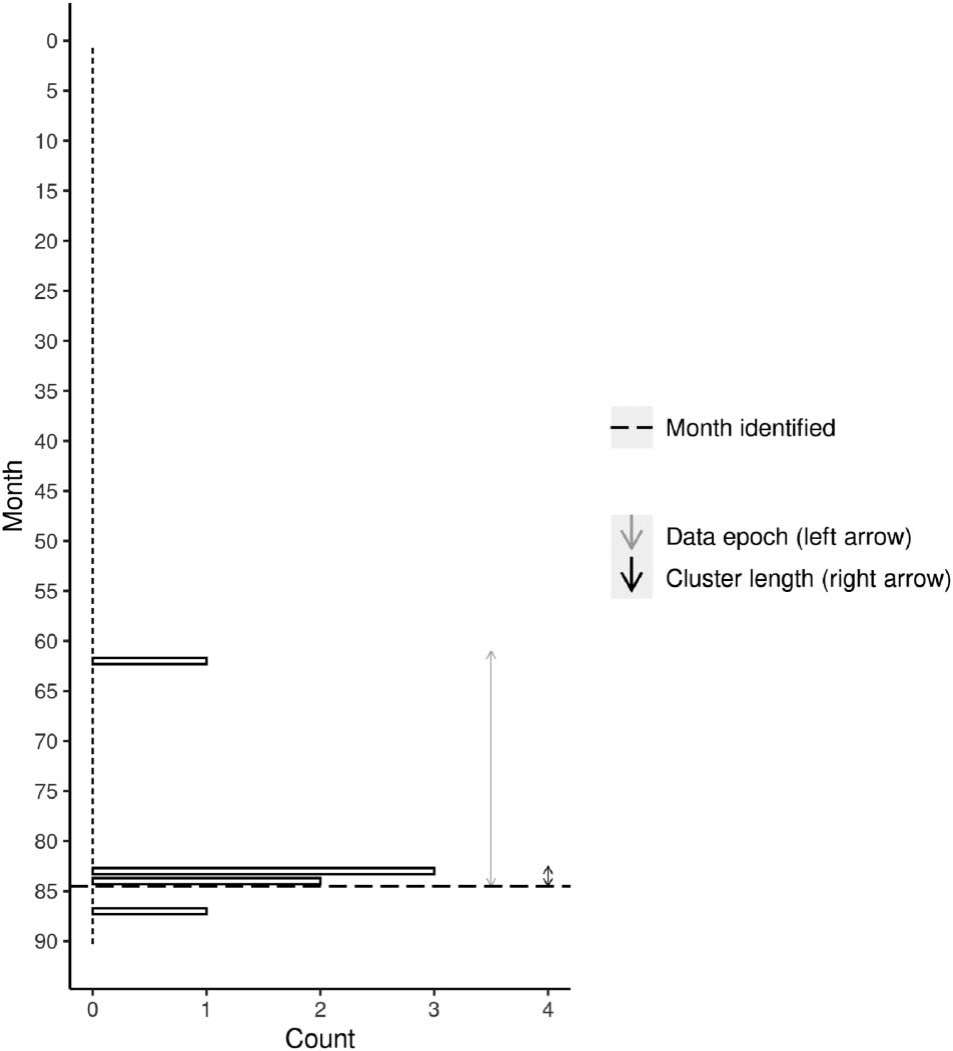
Timeline of cluster detection and suicide events for under-25 cluster 4c (5 suicides).

**Figure 4:**
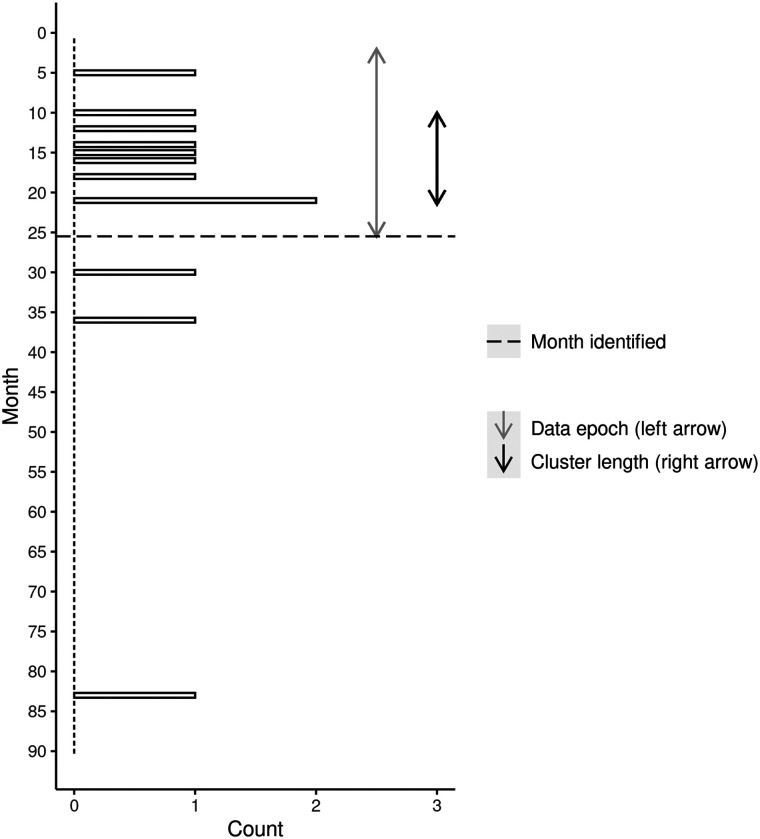
Timeline of cluster detection and suicide events for under-25 cluster 1a (8 suicides).

Table 2 summarizes the details of the earliest detection of each significant cluster identified from the under-25 simulation. In total, eight distinct significant clusters were identified, with duplication again observed in some technically distinct clusters (e.g., Cluster 4). Cluster detection timelines are presented in Figures 3 and 4, respectively, for Cluster 4c and Cluster 1a. Figure 3 demonstrates little history of suicide inside the cluster boundary until the cluster occurred between months 83 and 84. Our modeling indicates that this cluster would have been identified following supply of data of month 84. Figure 4 shows the occurrence of a cluster between months 10 and 21. Our modeling indicates that this cluster would have been identified following supply of data at the end of month 25.

**Table 2:** Significant clusters identified in the under 25-year-old analysis

Cluster	Geography level	Duration of cluster (months)	Number of areas in cluster	Observed	Expected	*p* value	Population of cluster area
1a	SA1	12	100	8	0.536	.083	1,200
2a	SA1	4	6	nr	0.009	>.01	>600
3a	MB	1	82	nr	0.006	>.01	>1,500
3b	SA1	1	18	nr	0.008	>.01	>2,000
3c	SA2	1	4	nr	0.045	>.01	>10,500
4a	SA1	3	156	6	0.211	.081	16,200
4b	SA1	5	156	7	0.338	.093	16,200
4c	SA2	2	6	5	0.131	.008	15,000
*Note*. Functionally equivalent clusters have been grouped (e.g., 4a – 3c), even if they have marginally distinct cluster dimensions. nr = not reported due to small cell sizes. Where this occurs, the *p* value and the population size have been rounded up to preserve the anonymity of the cluster’s location.							

## Discussion

We tested the feasibility of establishing a real-time suicide cluster monitoring system using data recorded in the VSR. Unlike traditional approaches to cluster detection, which rely on a single scan at a point in time, we designed a cluster detection system with the following key elements: (1) Scans were undertaken on a 2-year epoch of data. Each epoch was essentially a moving window of data, with the most recent month of data replacing the oldest month. (2) Multiple scans were undertaken on each epoch. Because the choice of spatial unit of analysis has the potential to influence the scan results, we fit models on the smallest available spatial unit (mesh blocks) through to a relatively large spatial unit that represents communities that interact together socially and economically (SA2s; Australian Bureau of Statistics, July 2021). (3) Scans were undertaken for all age groups and for people aged <25 years.

Our approach meant that multiple scans were undertaken on each epoch. The principle underlying this approach was that we wanted to identify clusters under a variety of settings known to influence the scanning results. Our results revealing the rapid detection of significant clusters demonstrates that monthly provision of suicide data does, in a place such as Victoria with around 700 suicides per year, allow the detection of suicide clusters in real time.

We have developed several different ways of presenting the results of our scans to those approved end users who have responsibility for responding to a cluster. Although not shown here, we are able to plot the location of all suspected suicides and the boundaries of all clusters in an interactive map where the user can zoom in and out of the map. Holding the mouse pointer over an individual case reveals further information such as the case number, age at death, and sex. This facilitates, for instance, a deeper file review of cases to identify commonalities between cases that may further indicate a cluster is in play. Our illustration of the cluster timeline (Figures 1–4) shows another way to present the results to end users. While the figures shown here have been deidentified, they are very similar to the identifiable versions developed for end users. These figures will also be useful for evaluating the success of any interventions that are deployed into these areas. A reduction in suicides would suggest that these interventions have been successful, while a continuation of the pattern observed during the cluster period would indicate they have not.

The implication of our findings is that it is feasible to combine a data recording system such as the VSR with the scan statistic to develop a real-time (or near real-time) suicide cluster detection system. Such an approach has two advantages over existing methods to detect clusters. Most obviously, it uses up to date data (unlike all previous studies in Australia), but equally importantly, it scans for clusters at a variety of different geographic levels minimizing the bias from the modifiable area unit problem. Conveniently, opensource software (e.g., R, rsatscan) can be applied to automate almost the entire cluster detection system, including processing data feeds, executing the scan statistic, and collating the results (e.g., reports, maps, and graphs).

Our study parallels an investigation conducted in Ireland (Benson et al., 2022), in which the researchers performed prospective Satscan analyses on a dataset containing information about 388 suicides. Unlike the Irish study, we identified a number of clusters (albeit at the *p* < .1 level), presumably on account of the greater number of suicides that we observed (e.g., approximately 1,300–1,400 all-age suicides per epoch). The Irish study demonstrated the feasibility of real-time cluster investigation using R packages with the notable strength that the authors developed a dashboard system for reporting their results to end users.

The system of scanning for clusters in space and time is likely to be useful for coroners. If coroners are made aware of an emerging suicide cluster, they can direct the focus of investigations into relevant cluster-implicated deaths from an early stage, creating better opportunities to collect information that may ultimately shed light on the cause and course of the cluster. Coroners need to balance competing considerations (for example, the deceased’s right to privacy, loved ones’ wishes, and public health imperatives) when determining whether to gather and/or share information about a death. They may be assisted in these considerations if they know the death is part of an emerging cluster and certain information could potentially assist in preventing further deaths.

### Limitations

Our simulation of a real-time suicide cluster detection system has limitations. Primarily, precise cluster detection with the scan statistic requires accurate population estimates. In Australia, estimates at smaller area levels are computed only at census times (every five years), potentially affecting the fidelity of cluster detection in intracensus years. An ideal for an effective suicide cluster alert system (using the scan statistic) is the automated supply of regular population data updates into the analysis.

Although we piloted numerous SaTScan options in arriving at our chosen settings, some variations remain that might improve cluster detection accuracy. One example is the use of ellipse-shaped windows (instead of the circular window setting), which could allow improved cluster detection in coastal regions, or along other natural, noncircular boundaries (Kulldorff et al., 2006). Another potential improvement would be to apply SaTScan’s space–time permutation model, which detects clusters without the need to aggregate events by area. Such an approach would allow detection of clusters without any boundary constraints and has been used in Wales (Jones et al., 2013) and more recently in Taiwan (Lu et al., 2023). We did not implement these approaches primarily because of the extensive computation burden (i.e., the time taken to fit these models meant it was not feasible in the context of real-time detection). We also fit our models in the retrospective mode. The prospective mode offers more precise *p* values due to adjustment for multiple analyses performed over time, although in practice it gave very similar results to what we have presented here.

### Conclusion

This feasibility study sought to understand if the application of the scan statistic to real-time suicide data with precise geocoordinates could be used to monitor the emergence of suicide clusters in a large Australian jurisdiction. Our findings show that this approach is highly feasible. Using data from a real-time suicide register, we were able to simulate a practical method of suicide cluster monitoring, that is, monthly scanning using rolling 2-year data epochs. We were able to identify a set of clusters with these data and automatically produce outputs illustrating their timing and location. These encouraging findings provide a platform to develop a live suicide cluster monitoring system in Victoria. More generally, the approach we have developed for detecting clusters could be applied to other jurisdictions both in Australia and internationally.

## Notes

**Publication Ethics:** Ethics approval was obtained from The University of Melbourne’s Human Research Ethics Committee (reference: 2021-21928-20627-3). The Victorian State Coroner signed an order for the release of coronial data for the purpose of this project and an information sharing agreement between the Court Services Victoria (acting on behalf of the Coroner’s Court of Victoria) and The University of Melbourne was executed in October 2021.

**Open Data:** The data used for this study were provided by the Coroners Court of Victoria. The authors are not permitted to share the data.
